# Anticancer drug response prediction integrating multi-omics pathway-based difference features and multiple deep learning techniques

**DOI:** 10.1371/journal.pcbi.1012905

**Published:** 2025-03-31

**Authors:** Yang Wu, Ming Chen, Yufang Qin

**Affiliations:** 1 College of Information Technology, Shanghai Ocean University, Shanghai, China; 2 Key Laboratory of Fisheries Information Ministry of Agriculture, Shanghai, China; Michigan State University, UNITED STATES OF AMERICA

## Abstract

Individualized prediction of cancer drug sensitivity is of vital importance in precision medicine. While numerous predictive methodologies for cancer drug response have been proposed, the precise prediction of an individual patient’s response to drug and a thorough understanding of differences in drug responses among individuals continue to pose significant challenges. This study introduced a deep learning model PASO, which integrated transformer encoder, multi-scale convolutional networks and attention mechanisms to predict the sensitivity of cell lines to anticancer drugs, based on the omics data of cell lines and the SMILES representations of drug molecules. First, we use statistical methods to compute the differences in gene expression, gene mutation, and gene copy number variations between within and outside biological pathways, and utilized these pathway difference values as cell line features, combined with the drugs’ SMILES chemical structure information as inputs to the model. Then the model integrates various deep learning technologies multi-scale convolutional networks and transformer encoder to extract the properties of drug molecules from different perspectives, while an attention network is devoted to learning complex interactions between the omics features of cell lines and the aforementioned properties of drug molecules. Finally, a multilayer perceptron (MLP) outputs the final predictions of drug response. Our model exhibits higher accuracy in predicting the sensitivity to anticancer drugs comparing with other methods proposed recently. It is found that PARP inhibitors, and Topoisomerase I inhibitors were particularly sensitive to SCLC when analyzing the drug response predictions for lung cancer cell lines. Additionally, the model is capable of highlighting biological pathways related to cancer and accurately capturing critical parts of the drug’s chemical structure. We also validated the model’s clinical utility using clinical data from The Cancer Genome Atlas. In summary, the PASO model suggests potential as a robust support in individualized cancer treatment. Our methods are implemented in Python and are freely available from GitHub (https://github.com/queryang/PASO).

## Author summary

Cancer treatment outcomes vary significantly among patients, presenting a critical challenge in personalized medicine. In this study, we developed PASO, a deep learning model that predicts how cancer cells respond to anticancer drugs. Unlike traditional approaches that focus on individual genes, we concentrated on the interactions between various molecules through biological pathways, combining these pathway-level features with drug chemical structures as model input features. By employing multiple deep learning techniques like transformer encoder, multi-scale convolutional networks and attention mechanisms, PASO captures complex interactions between drugs and cancer cells more effectively than existing methods. Compared with the state-of-the-art methods, our model achieved the best performance, meanwhile providing interpretable insights into the biological pathways and drug structures that influence treatment outcomes. Our model demonstrated particular success in identifying effective drugs for different cancers, such as PARP inhibitors in lung cancer. Through validation with clinical data from The Cancer Genome Atlas, PASO shows promising potential to guide personalized cancer treatment decisions, as demonstrated by its accurate prediction of clinical responses and significant correlation with patient survival outcomes.

## Introduction

Cancer is a highly heterogeneous and complex disease, stemming from the significant variabilities in the tumoral and its surrounding microenvironment at histopathological, genomic, and transcriptomic levels [1]. This heterogeneity in cancer leads to varying patient responses to the same treatment, as well as notable variances in prognostic outcomes, making personalized treatment for patients of vital importance. Clinical experiments such as the Cell Culture Drug Response Assay, though insightful for guiding treatment choices, are cost-prohibitive and often limited by sample volume [2]. It is essential to use computational methods to predict drug sensitivity for patients on a large-scale data. However, even within the same cancer type, there can be a clear individual variation in the effectiveness of the same treatment at the individual level, making the accurate prediction of a cancer patient’s sensitivity to anticancer drugs a challenge.

With the establishment of large-scale pharmacogenomic databases and the rapid advancement of machine learning and deep learning techniques, precise prediction of anticancer drug sensitivity has become possible. The Cancer Genome Atlas (TCGA) [3] and the Cancer Cell Line Encyclopedia (CCLE) [4] have collected multi-omics data from patients and cell lines of various cancer types, while the Genomics of Drug Sensitivity in Cancer (GDSC) [5] database provides information on the drug responses (measured by half-maximal inhibitory concentration, IC50) of nearly 1,000 cancer cell lines to approximately 500 anticancer drugs. Based on these databases, many machine-learning-based drug response models have been proposed. Lindsay C Stetson et al. [6] evaluated the performance of three classic machine learning algorithms Support Vector Machines (SVM), Random Forests (RF) and Elastic Net in predicting drug responses. The results showed that the predictive performance of RF and SVM was superior to that of Elastic Net regression models. Sakellaropoulos T. et al. [7] reported deep neural network models for drug response prediction based on gene expression data, outperforming traditional machine learning algorithms such as Elastic Net and Random Forest. Joo et al. [8] introduced the DeepIC50 model, which predicts the sensitivity of anticancer drugs using Convolutional Neural Network (CNN) technology and integrates the genetic mutation characteristics of cancer cells with the molecular fingerprint information of drugs. Differing from the former study that used gene mutation data, the MOLI model developed by Nguyen G.T.T. et al. [9] integrated multiple omics data including somatic mutations, copy number variations, and gene expression data for drug sensitivity prediction. Multimodal architectures have been demonstrated increasingly significant in precision medicine. Manica et al. [10] proposed the PaccMann model, which integrates drug molecular structure sequences, gene expression profiles, and protein-protein interaction networks, and improved both the accuracy and interpretability of drug sensitivity prediction through various deep learning techniques. Liu et al. devised the GraphCDR [11] model, which combines genomic mutation, gene expression, DNA methylation and molecular structural graph data of drugs, aiming to enhance the accuracy and generalizability of drug sensitivity predictions. Chen et al. developed scDEAL [12], which integrates bulk and single-cell RNA-seq data through deep transfer learning to predict drug responses at the single-cell level. Furthermore, the MM-Net framework [13] integrates gene expression data, whole-slide histology images, and drug molecular descriptors to predict drug responses in patient-derived xenograft tumors. By leveraging biological pathway prior knowledge, Ammad-Ud-Din, Muhammad, et al. [14] developed cwKBMF (component-wise Kernelized Bayesian Matrix Factorization), a method that integrates this prior knowledge with genomic data through multiple kernel learning. Next, the Precily model developed by Chawla et al. [15], utilized pathway activity estimates and drug descriptors as features to predict the drug sensitivity of cancer cells. Similarly, Tang and Gottlieb developed PathDSP [16], which performs enrichment analysis on cancer signaling pathways through multiple genomic data types and combines these pathway-level features with Morgan fingerprints of drug structures for drug sensitivity prediction. Recently, Zhang et al. introduced HiDRA [17], a hierarchical attention network that combines pathway information with drug features represented by Morgan fingerprints for drug response prediction, enhancing interpretability and accuracy.

Although these models have made significant progress in drug response prediction, several limitations are still observed. On one hand, most studies (such as [6–11]) primarily rely on single-gene level features, without fully considering the functional associations between genes. While cwKBMF [14] and Precily [15] attempted to incorporate pathway information, they overlook the interpretability of pathways in drug response prediction. On the other hand, regarding drug feature representation, existing methods such as DeepIC50 [8], PathDSP [16], and HiDRA [17] utilize molecular fingerprints, while Precily [15] and MM-Net [13] employ drug descriptors, which struggle to comprehensively capture the chemical structural information of drugs, thus limiting the in-depth understanding of drug mechanisms of action. We attempt to overcome these limitations through the following aspects. First, unlike methods using single-gene level features, we capture pathway-level biological changes by computing the differences in multi-omics data within and outside pathways. As is well known, biological pathways describe the interactions between various molecules within cells, reflecting the regulatory mechanisms of many key physiological processes. Most targeted therapies exert their effects through specific biological pathways and the essence of biology will be overlooked without considering the role of pathways [18]. Secondly, we adopt a multi-scale drug feature extraction framework to thoroughly mine the chemical structural information of drugs, providing a more comprehensive description of drug molecular features. Furthermore, we utilize attention mechanisms to learn the interactions between drug features at different scales and omics features. This network can assign attention weights to each chemical molecule in the SMILES sequence and each biological pathway, thereby enabling the assessment of their contributions to drug response prediction.

Based on the considerations mentioned above, we propose a drug response model, which utilizes features of the differences between within and outside of biological pathways in multi-omics data, as well as the SMILES chemical structure features of drugs with an attention mechanism for calculating SMILES-Omics interactions (referred as PASO), to predict the response of cell lines to drugs. We collected drug response information of cell lines from the GDSC2 dataset and drug SMILES information from PubChem. The gene expression data, gene copy number variation data, and mutation data of the cell lines were downloaded from CCLE. The drug SMILES information was first processed into a uniform length of digital encoding with pytoda. Then, utilizing the 619 KEGG_MEDICUS pathway gene sets in the MSigDB database, we calculated pathway-based difference values for gene expression data in 688 pre-screened cell lines using the Mann–Whitney U test [19] and for gene copy number variation data as well as mutation data using the Chi-square-G test, resulting in three types of pathway difference value encoded omics data. We adopted an Embedding network, multiscale convolutional neural networks, and transformer encoder to re-encode SMILES drug information, providing multi-perspective representations of drug chemical structural features. Attention mechanism networks were used to handle the interactions between drug and omics data, assigning attention weights to different pathways and chemical structures. Next, we considered omics features and drug features as explanatory variables and the drug response value LN IC50 as the dependent variable for regression tasks, using these pre-processed features to predict drug sensitivity. Finally, the model’s generalization ability was validated through tenfold cross-validation and compared with classical machine learning algorithms and recently proposed deep learning models, demonstrating its superior predictive performance. We further validated the model’s application using TCGA clinical data, where the model not only accurately predicted patient drug responses but also showed significant correlation with patient prognosis. Overall, the PASO model demonstrates superior predictive performance and interpretability, while showing significant potential in clinical applications.

## Result

### Performance evaluation of PASO in predicting drug sensitivity

To comprehensively evaluate the performance of the proposed PASO, we implemented three data splitting strategies: (i) Mixed-Set, (ii) Cell-Blind, and (iii) Drug-Blind [20]. The model’s performance was assessed using three different evaluation metrics: mean squared error (MSE), Pearson’s correlation coefficient (PCC), and the coefficient of determination (R²), which are commonly used for evaluating the performance of regression models. Furthermore, to assess the model’s generalization ability, we performed tenfold cross-validation, and validated the model using different combinations of omics data as input.

**Fig 1:**
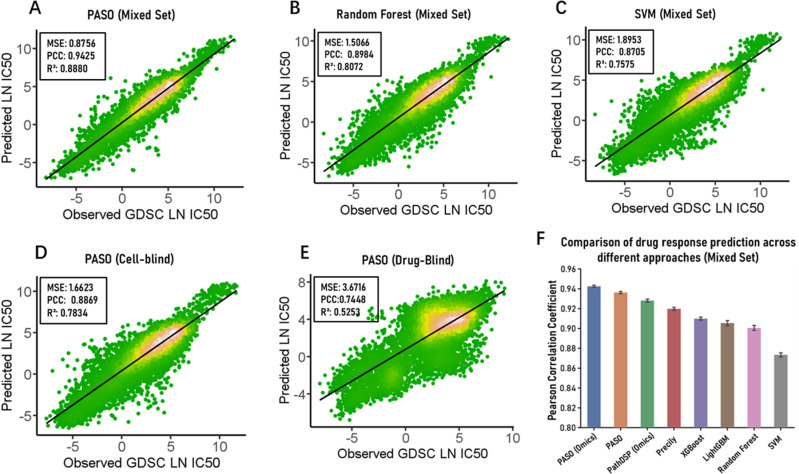
Performance observation of the PASO model (A-C) comparison of PASO with classical machine learning models Random Forest and SVM. For each machine learning model, the same features (619 pathway differential features and 256-dimensional drug SMILES digital encoding) were used as input. (D-E) Predictive performance of PASO under Cell-Blind and Drug-Blind conditions, respectively. (F) The bar chart shows the comparison of drug response prediction among different approaches. Approaches with the ‘Omics’ suffix indicate the use of three types of omics data (Gep, CNV, and Mut).

For a rigorous comparative evaluation, we tested PASO and all baseline methods on the same dataset, which contains 141,222 cell line-drug pairs, comprising 688 pre-screened cell lines and 233 drugs (See the Data preparation in Method section). Under the Mixed-Set splitting strategy, 10% of the data was used as the test set, while the remaining data was used for model training. On the test set, the model exhibited outstanding performance (Fig 1A), with an MSE of 0.8756, a PCC of 0.9425, and an R² of 0.8880. Under this strategy, we compared the model’s performance with traditional machine learning methods, Random Forest (RF) [21] and Support Vector Machines (SVM) [22], using the same training and test sets, with gene expression pathway-based difference values of cell lines and numerical encodings of drug SMILES as inputs. The results showed that, in terms of MSE, the model achieved reductions of 41.88% and 53.80% compared to RF and SVM, respectively. Regarding PCC, the model outperformed RF by 4.91% and SVM by 8.27% (Fig 1B and 1C). Therefore, PASO significantly outperformed these two classic machine learning algorithms.

We further evaluated the performance of PASO under the Cell-Blind and Drug-Blind approaches because, in the Mixed-Set method, the training and test sets contain shared cell lines and drugs, which allows the model to learn specific omics features and drug sensitivity information of some cell lines in the test set during training, making it relatively easy to predict the responses of these cell lines to new drugs. Actually, in clinical applications for new patients, we cannot anticipate their responses to any drugs beforehand. Therefore, we adopted the Cell-Blind Set method to ensure that the cell lines in the training and test sets were mutually exclusive, better reflecting the clinical reality. Under the Cell-Blind strategy, the model achieved an MSE of 1.6623, a PCC of 0.8869, and an R² of 0.7834 (Fig 1D). Although the model’s accuracy decreased compared to the Mixed-Set, it still maintained good performance, indicating that even in practical clinical scenarios where cell line features are unknown, the model yielded reliable predictions. Furthermore, under the Drug-Blind strategy, predicting the responses of known cell lines to new drugs better simulates the situations encountered during drug development in the real world. We ensured that the drugs in the test set were not present in the training set, preventing the model from relying on specific drug response information learned during the training phase for prediction, further increasing the challenge. Under this strategy, the model achieved an MSE of 3.6716, a PCC of 0.7448, and an R² of 0.5253 (Fig 1E), still maintaining considerable predictive accuracy. Compared to the Cell-Blind approach, the model’s accuracy further decreased, indicating that predicting the responses to unknown drugs poses a greater challenge than predicting new cell lines [20].

We also utilized the pre-screened 141,222 cell line-drug pairs and applied a tenfold cross-validation method to evaluate the impact of different omics data combinations on the prediction results and the model’s generalization. Under the Mixed-Set approach, we compared the cases where all three omics data were used as input with using only gene expression (denoted as GEP) and combinations of gene expression with other omics data (e.g., GEP&MUT) as input. Since some studies have claimed that gene expression (the transcriptomic feature of cell lines) is the most powerful feature in predicting cancer drug response [23,24], we took gene expression features as the basis and explored the effects of combining them with other omics features. The results (S3 Fig) showed that when using only gene expression data as input, the MSE was the highest. When using the combination of gene expression and other omics data as input, the MSE decreased relatively, and the results also became more stable. However, when using all three omics data as input, the MSE was the lowest, and the results were also relatively stable. This demonstrated that the model could learn more biological information from the additional omics data. The pathway-based difference values of gene copy number variation and gene mutation obtained through the Chi-square-G test method (see Methods section) significantly improved the model’s predictive ability, thus proving the effectiveness of our omics data preprocessing method in promoting the model’s learning.

To comprehensively evaluate PASO’s performance, we conducted tenfold cross-validation using a Mixed-Set splitting strategy on a dataset of 141,222 cell line-drug pairs. The comparative evaluation included four classical machine learning methods (Random Forest, SVM, LightGBM, and XGBoost) and two recently proposed deep learning models (Precily and PathDSP). All machine learning methods utilized 619 gene expression pathway difference features and 256-dimensional drug SMILES digital encodings as input features. For deep learning model evaluation, we followed the feature selection strategies from their original studies. Precily originally used only gene expression and drug SMILES features for prediction, so we maintained this feature combination for comparison. PathDSP originally employed gene expression, somatic mutations, copy number variations, drug targets, and Morgan fingerprints as input features. Since this approach aligns with our multi-omics data methodology, we used 619 pathway difference features for each of the three types of omics data (GEP, CNV, Mut) combined with 256-dimensional drug SMILES encoding for comparison. This choice both ensured fairness in comparison and adhered to the original design philosophies of each model. To ensure fair comparison, all deep learning models were implemented in Pytorch (https://pytorch.org/), and we employed the Optuna library (https://optuna.org/) for hyperparameter optimization across all deep learning models, including key parameters such as number of hidden layers, neuron counts, dropout rates, and learning rates. Each model underwent 50 trials with different hyperparameter combinations, with the optimal configuration selected based on validation loss minimization (optimal hyperparameters). In the cross-validation experiments, we uniformly set the maximum training epochs to 200 and fixed the batch size at 512, with detailed hyperparameter descriptions provided in S1 Table. For our proposed PASO, we conducted two sets of experiments: one using only gene expression data, and the other using all three types of omics data. The cross-validation results, as shown in Fig 1F, demonstrate that PASO utilizing three types of omics data achieved the best performance (PCC = 0.9425), followed by the PASO version using only gene expression data (PCC = 0.9372). PathDSP, leveraging multi-omics data, achieved the third-best performance (PCC = 0.9282), while Precily, using the same input features as other machine learning methods, performed well (PCC = 0.9198), outperforming other machine learning models. Among traditional machine learning methods, modern ensemble learning approaches XGBoost (PCC = 0.9100) and LightGBM (PCC = 0.9054) outperformed conventional Random Forest (PCC = 0.9006) and SVM (PCC = 0.8735). Additional detailed performance metrics are presented in Table 1. These results strongly validate PASO’s superior performance in drug sensitivity prediction tasks, particularly demonstrating significant advantages when integrating multi-omics data for prediction.

**Table 1:** Comparison of drug response prediction across different approaches.

Model name	RMSE (±sd)	PCC (±sd)	R² (±sd)
SVM (Gep, Smi)	1.3632 (±0.0088)	0.8735 (±0.0021)	0.7630 (±0.0036)
Random Forest (Gep, Smi)	1.2171 (±0.0103)	0.9006 (±0.0025)	0.8110 (±0.0044)
LightGBM (Gep, Smi)	1.1953 (±0.0079)	0.9054 (±0.0026)	0.8178 (±0.0032)
XGBoost (Gep, Smi)	1.1611 (±0.0066)	0.9100 (±0.0016)	0.8280 (±0.0029)
Precily (Gep, Smi)	1.1011 (±0.0095)	0.9198 (±0.0016)	0.8311 (±0.0042)
PathDSP (Gep, CNV, Mut, Smi)	1.0499 (±0.0154)	0.9282 (±0.0015)	0.8365 (±0.0116)
PASO-Non-Attention (Gep, Smi)	1.0059 (±0.0450)	0.9333 (±0.0066)	0.8575 (±0.0189)
PASO (Gep, Smi)	0.9882 (±0.0120)	0.9363 (±0.0012)	0.8709 (±0.0025)
**PASO (Gep, CNV, Mut, Smi)**	**0.9400 (±0.0081)**	**0.9425 (±0.0011)**	**0.8838 (±0.0021)**

Gep denotes gene expression profiles; Mut represents somatic mutation data; CNV indicates copy number variation; Smi refers to the SMILES representation of drugs.

Finally, we designed a controlled experiment to evaluate the impact of incorporating the attention module on model performance. Specifically, a non-attention version of the model (PASO-Non-Attention) was constructed, where the original attention module was replaced with a deep neural network (DNN) while keeping the overall model architecture unchanged. The attention module in the original PASO model is responsible for capturing the interactions between multi-scale SMILES drug information and omics data. In the non-attention version, these interactions were learned using a DNN, and feature fusion was performed in the final MLP layer, resulting in a black-box structure. This modified model utilized the same input features as the original PASO model, including 619 pathway difference features for gene expression and 256-dimensional SMILES encodings. The performance evaluation results (see Table 1) demonstrate that our PASO model outperformed the non-attention version across all metrics. This indicates that incorporating the attention module not only enhanced the model’s interpretability but also improved its predictive accuracy.

### Prediction and analysis of lung cancer cell lines

Lung cancer is one of the malignant tumors with the highest incidence and mortality rates globally. Based on its histological and cytological characteristics, lung cancer is primarily categorized into two main types: non-small cell lung cancer (NSCLC) and small cell lung cancer (SCLC). NSCLC can be further subdivided into subtypes such as adenocarcinoma (LUAD), squamous cell carcinoma (LUSC), and large cell carcinoma (LCLC), with LUAD and LUSC being the primary NSCLC subtypes. Small cell lung cancer (SCLC) can be classified into limited-stage and extensive-stage based on the extent of dissemination. To simplify the research, we do not further subclassify it [25].

**Fig 2:**
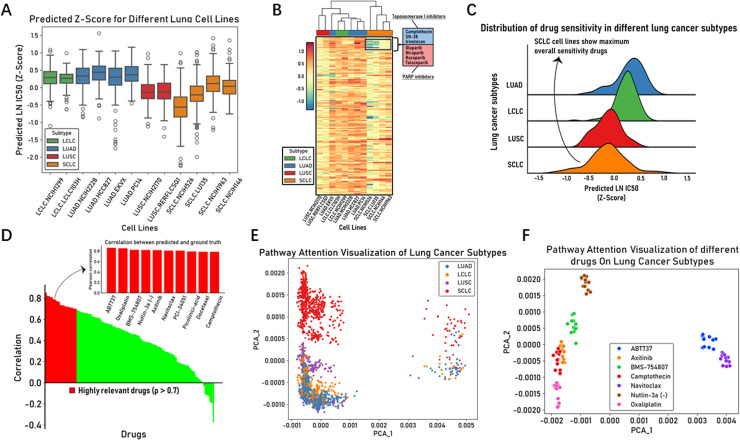
Comprehensive analysis of lung cancer cell lines (A) Box plot depicting the LN IC50 (Z-score) values predicted for all drugs across each lung cancer cell line. (B) Heatmap showing the predicted LN IC50 (Z-score) for each drug in each lung cancer cell line. (C) Ridge plot displaying the overall distribution of predicted LN IC50 (Z-scores) across the four lung cancer subtypes. (D) Waterfall chart showing the ranking of predictive performance for each drug. The red portion is used to differentiate drugs with high accuracy (ρ>0.7). The bar plot displays the top 10 drugs with highest accuracy. (E) PCA plot showing the clustering result of pathway-level attention weights based on different lung cancer subtypes. (F) PCA plot showing the clustering result of pathway-level attention weights for these lung cancer subtypes based on different drugs.

In the well-trained PASO model shown in Fig 2D, we selected 12 different types of lung cancer cell lines from the test set, including EKVX, NCIH2228, HCC827, and PC14 for LUAD; NCIH2170 and RERFLCSQ1 for LUSC; LCLC-103H and NCIH1299 for LCLC; and NCIH526, LU-35, NCIH146, and NCIH1963 for SCLC, to observe the model’s predictive performance on these cell lines. We predicted the LN IC50 values of the corresponding drug responses for each cell line and calculated the Z-scores of LN IC50 (Fig 2A and 2B), as Z-scores help identify and compare the relative drug sensitivity, allowing us to intuitively observe their statistical deviations from the average level. Among these twelve lung cancer cell lines, NCIH526 exhibited the highest drug sensitivity (Fig 2A). In a study on drug screening for small cell lung cancer (SCLC) [26], NCIH526 was also listed as one of the most sensitive SCLC cell lines. The heatmap shows that SCLC cell lines have potential sensitivity to Topoisomerase I inhibitors and PARP inhibitors (Fig 2B). Specifically, Topoisomerase I inhibitors include Camptothecin, SN-38, and Irinotecan, while PARP inhibitors cover Olaparib, Niraparib, Rucaparib, and Talazoparib, among which Talazoparib demonstrated the highest potential sensitivity across all drugs. This aligns with recent studies, as some clinical trials have shown that PARP inhibitors exhibit potent single-agent activity in SCLC cell lines, particularly when combined with DNA-damaging agents [27,28]. Camptothecin and its derivatives (Irinotecan and SN-38), as Topoisomerase I inhibitors and a class of DNA-damaging agents, have been reported to show certain efficacy in treating SCLC at an early stage [29–31]. Interestingly, both Topoisomerase I inhibitors and PARP inhibitors exert their anti-tumor effects by interfering with key steps in the DNA replication process, disrupting normal DNA replication and repair. We noticed that the difference values of the DNA replication pathway in the gene expression data of SCLC cell lines were significantly higher than those of other pathways (S1 Fig), which might explain the predicted sensitivity of these four SCLC cell lines to these drugs. Additionally, the average Z-scores for LUAD, LCLC, LUSC, and SCLC lung cancer subtypes in the ridge plot were 0.326, 0.256, -0.135, and -0.152, respectively (Fig 2C). Among these four lung cancer subtypes, SCLC exhibited the highest drug sensitivity. As a class, SCLC is usually more sensitive to chemotherapy and radiation therapy than NSCLC [32].

We ranked all drugs acting on the 12 lung cancer cell lines in descending order based on their predicted Pearson’s correlation coefficients for drug responses (Fig 2D). Among the top ten drugs, chemotherapeutic agents targeting DNA replication (e.g., Oxaliplatin) and mitosis (e.g., Docetaxel) were included, as well as targeted drugs affecting apoptosis regulation (e.g., ABT737 and Navitoclax) and IGF1R signaling (e.g., BMS-754807). This may suggest that the selection of drugs for lung cancer treatment should consider the diversity of mechanisms of action, encompassing both traditional chemotherapeutic agents and emerging targeted therapies. Furthermore, ABT737, BMS-754807, Nutlin-3a (-), Oxaliplatin, Navitoclax, Docetaxel, and Camptothecin have demonstrated therapeutic or potential therapeutic effects in lung cancer [31,33–38], and the pathways targeted by these drugs are also diverse. Our method revealed the importance of considering the diversity of drug target pathways in lung cancer treatment, a diversity that manifests not only between chemotherapeutic and targeted agents but also among drugs targeting different molecular mechanisms of action.

Next, we investigated the pathway attention scores of these cell lines, leading to an unexpected finding that they can reveal the importance of various biological pathways within the omics features. We performed principal component analysis (PCA) on the pathway attention scores of the four lung cancer subtypes, mapping the pathway attention scores onto a two-dimensional plane to study whether the pathway-level attention scores reflect the tumor characteristics of the cell lines. Interestingly, PCA nicely separated the cell line-drug pairs of the two major lung cancer subtypes (NSCLC and SCLC) (Fig 2E), indicating that the pathway attention scores effectively captured the cancer types of the cell lines. Different lung cancer subtypes (NSCLC and SCLC) exhibit significant differences in biological pathways and drug responses, and these differences can be captured by the model and used to distinguish tumor characteristics. However, for the three subtypes within NSCLC, they shared substantial overlap in their drug response data due to similarities in their biology and treatment approaches. Furthermore, we noticed that some points were distributed at PCA_1 > 0.002, and we investigated the cell line-drug response information corresponding to these points, and contrasted the chemical properties of these drugs with those of drugs at PCA_1 < 0.002. Excitingly, the chemical properties of these drugs differed significantly from those of drugs at PCA_1 < 0.002. Specifically, significant differences were observed in properties such as MolWt (molecular weight), TPSA (topological polar surface area), MolLogP, and NumHDonors (S2 Fig), which are related to cellular permeability, water solubility, lipophilicity, and the number of hydrogen bond donors of the drugs [39]. This suggests that the chemical properties of drugs interact with the pathway features of cell lines, and the model can capture these interactions and utilize them to distinguish the chemical properties of drugs. In other words, there exist interactions between drug features and pathway attention scores, which corresponds to the model structure we employed: the SMILES&Omic Attention Layer (see Methods), which can compute the interactions between cell line features and drug features. Since there are interactions between drugs and cell lines, we further selected the seven most accurately predicted drugs for the four lung cancer subtypes and again used PCA to map the pathway attention scores onto a two-dimensional plane, coloring each data point according to different drugs. We found that each drug was distinctly separated (Fig 2F), implying that the model can capture the differences in chemical properties of various drugs involved in the cell line-drug response process. This further demonstrates that the trained PASO can learn the complex interactions between the omics data of cell lines and drugs.

### Overall analysis of pathway attention weights

**Fig 3:**
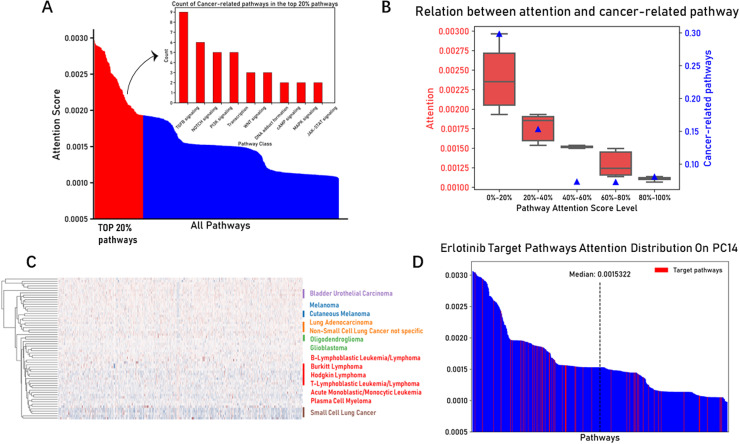
Overall analysis of pathway attention weights (A) Waterfall chart showing the ranking of average attention scores across all pathways. The red portion is used to differentiate the top 20% of pathways. The bar plot displays the 9 pathways most closely related to cancer development and progression among the top 20%. (B) Pathway attention scores are divided into five groups by proportion. The red box plot shows the distribution of pathway attention scores in each group, and the blue triangles indicate the proportion of pathways closely related to cancer development and progression. (C) Hierarchical clustering result of pathway attention scores, with many pathways of the same or similar cancer types clustered together. (D) Waterfall chart showing the distribution of the ranking of the targeted pathway of the drug Erlotinib among all pathway scores.

The advantage of PASO is its interpretability, which mainly stems from the model’s attention mechanism. In the above lung cancer analysis, we learned that the model’s attention mechanism can capture the differences in biological pathways and drug responses across different lung cancer subtypes, and it can be used to distinguish tumor characteristics. Furthermore, we analyzed the pathway attention scores for all cell lines. We calculated the average attention score for each pathway in the test set cell lines and ranked the pathways from high to low based on their attention scores. We then counted the number of cancer-related pathways among the top 20% of pathways with the highest attention scores (Fig 3A). Nine pathways were identified closely associated with cancer: TGFB signaling, PI3K signaling, NOTCH signaling, MAPK signaling, WNT signaling, DNA adduct formation, Transcription, JAK-STAT signaling, and cAMP signaling. These signaling pathways precisely regulate crucial life activities such as cell proliferation, apoptosis, differentiation, and migration under normal physiological conditions. While their abnormal activation or inhibition in pathological tumor states serves as a key driving force for uncontrolled cellular behavior and malignant transformation. The results showed that among the top-ranked pathways by attention scores, the TGFB, PI3K, and Notch signaling pathways were more prevalent, and these pathways are closely associated with cancer development and progression. The TGFB signaling pathway plays multifaceted roles in cell proliferation, differentiation, apoptosis, and intercellular interactions, capable of both suppressing tumor growth and promoting the formation of the tumor microenvironment [40]. The PI3K signaling pathway is a key regulator of cell survival, metabolism, and proliferation, and its activation is often linked to cancer progression [41]. The NOTCH signaling pathway, as a crucial regulator of cell fate determination, plays a central role in cell differentiation, development, and various cancers [42]. To better observe the distribution of cancer-related pathways, we divided all pathways into five groups based on their attention scores from high to low. The red box plots show the distribution of pathway attention scores for each group, while the blue triangles indicate the proportion of cancer-related pathways (Fig 3B). Interestingly, the proportion of cancer-related pathways was higher in the groups with higher attention scores. This suggests that the model prioritized cancer-related pathways when allocating attention scores.

Next, we calculated the average attention score of each pathway for every cell line sample and performed hierarchical clustering analysis on the pathway attention scores across all samples. Interestingly, cancer types of the same or similar nature were clustered together (Fig 3C). For instance, various blood cancers, including B-Lymphoblastic Leukemia/Lymphoma, Burkitt Lymphoma, Hodgkin Lymphoma, and T-Lymphoblastic, were clustered together. Similarly, Small Cell Lung Cancer (SCLC) and Non-Small Cell Lung Cancer (NSCLC) were clustered into distinct subgroups. Furthermore, Oligodendroglioma and Glioblastoma, two subtypes of neuroglial tumors, were also grouped together. The results demonstrated that pathway attention scores clustered according to tumor types. Additionally, we further displayed the distribution of targeted pathways for specific drugs in specific cell lines (Fig 3D). Taking the Erlotinib drug acting on the PC14 cell line as an example, over 60% of Erlotinib’s targeted pathways were distributed before the median of the pathway attention score distribution for all pathways in the PC14 cell line. This finding revealed the model’s ability to identify and evaluate drug mechanisms of action, specifically manifested in its capacity to capture and emphasize biological pathways directly related to drug effects.

Overall, the PASO model effectively highlighted signaling pathways closely related to tumor biology, such as TGFB, PI3K, and NOTCH, through its attention mechanism, allocating higher attention scores to these pathways and demonstrating excellent interpretability. Further analysis revealed that the proportion of cancer-related pathways was higher in the groups with higher attention scores, indicating that the model can automatically identify and emphasize relevant cancer pathways based on the tumor features of the cell lines. Additionally, the model confirmed the difference of pathway attention scores among cell lines through hierarchical clustering analysis, enabling the differentiation of various tumor types and the identification and emphasis of signaling pathways closely associated with drug targeting mechanisms.

### Drug efficacy prediction analysis and drug molecular weight analysis for targeted

In drug sensitivity prediction studies, predicting the responses to traditional chemotherapeutic agents is relatively straightforward, but predicting the responses to specific targeted drugs is more challenging [43]. This is because targeted drugs typically exhibit superior efficacy and selectivity only in tumor cell lines that have aberrations in the specific signaling pathways they target. In this study, we selected two drugs, Erlotinib and Refametinib. Erlotinib targets the EGFR signaling pathway by competitively inhibiting the tyrosine kinase activity of the receptor, thereby blocking cell proliferation [44]. Refametinib, on the other hand, targets the ERK/MAPK signaling pathway and is a highly selective MEK1/2 inhibitor. It inhibits the kinase activity of MEK1/2, thus blocking the downstream phosphorylation and activation of ERK1/2, ultimately inhibiting tumor cell proliferation, differentiation, and other processes mediated by the ERK/MAPK pathway [45,46].

**Fig 4:**
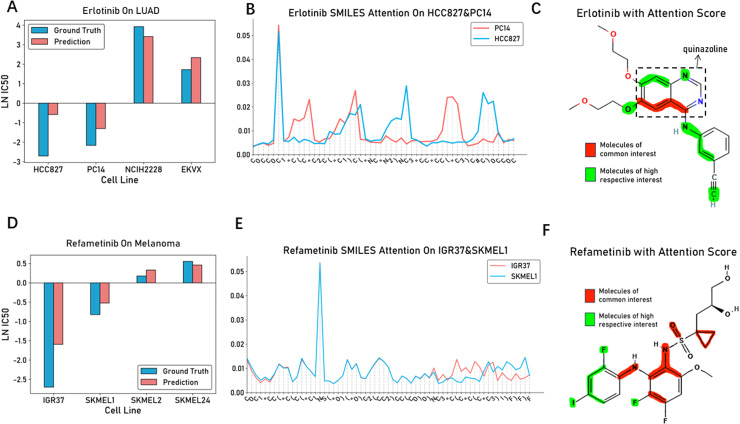
Drug efficacy prediction analysis and drug molecular weight analysis for targeted drugs (A-C) Bar plots showing the predicted ln IC50 values compared to observed values of Erlotinib in four LUAD cell lines; line plots showing the distribution of drug molecular attention weights of Erlotinib in PC14 and HCC827 cell lines; molecular weight visualization analysis for Erlotinib highlights molecular structures with attention scores > 0.01, using red to indicate molecular structures commonly attended to by cell lines, and green to indicate molecular structures individually attended to by each cell line. (D-F) Bar plots showing the predicted ln IC50 values compared to observed values of Refametinib in four LUAD cell lines; line plots showing the distribution of drug molecular attention weights of Refametinib in IGR37 and SKMEL1 cell lines; molecular weight visualization analysis for Refametinib highlights molecular structures with attention scores > 0.01, using red to indicate molecular structures commonly attended to by cell lines, and green to indicate molecular structures individually attended to by each cell line.

Firstly, we showcase the drug response prediction of Erlotinib on four lung adenocarcinoma (LUAD) cell lines (HCC827, PC14, NCIH2228, and EKVX) (Fig 4A). To clearly demonstrate the prediction accuracy, we present a comparison between the predicted LN IC50 values and the observed ground truth values. It can be observed that the model accurately predicted the drug response in these four cell lines. According to the Expasy database [47], both HCC827 and PC14 cell lines harbor the classic EGFR 19 exon deletion mutation (p.Glu746_Ala750del). This mutation enhances the tyrosine kinase activity of EGFR, leading to sustained activation of downstream tumor-associated signaling pathways and serving as a key driving event in the development of LUAD [48,49]. Consequently, HCC827 and PC14 are expected to exhibit higher sensitivity to the EGFR tyrosine kinase inhibitor Erlotinib, which is indeed reflected in their lower ground truth LN IC50 values. Similarly, we also showcase the drug response prediction of Refametinib on four melanoma (MEL) cell lines (IGR37, SKMEL1, SKMEL2, and SKMEL24) (Fig 4D), and achieved promising prediction results. The BRAF V600E mutation found in IGR37 and SKMEL1 cell lines is frequently observed in various melanomas (MEL) and leads to enhanced kinase activity of the BRAF protein, resulting in sustained activation of the MAPK/ERK signaling pathway, promoting tumor cell proliferation and survival [50]. Consequently, IGR37 and SKMEL1 cell lines exhibit higher sensitivity to Refametinib, and PASO accurately predicted the LN IC50 values of Refametinib on these four MEL cell lines.

Given the promising drug response of Erlotinib on HCC827 and PC14 cell lines, we further analyzed the attention weight distribution allocated by the model to the molecular structure of Erlotinib when predicting these two cell lines (Fig 4B). We highlighted the molecular substructures with attention scores > 0.01, using red color to represent the substructures commonly attended to by both cell lines and green color to represent the substructures attended to by each cell line individually (Fig 4C). The mechanism of action of Erlotinib is based on its quinazoline ring structure, which plays a crucial role in the ATP-binding pocket of its target, the epidermal growth factor receptor (EGFR) [51]. Our study found that for both HCC827 and PC14 cell lines, the model highly attended to three carbon atoms within the quinazoline ring of Erlotinib: one shared carbon atom inside the ring and two adjacent carbon atoms, one connected to a (3-ethynylphenyl)amino group and the other to a methoxyethoxy group. The (3-ethynylphenyl)amino group of Erlotinib forms stable hydrogen bonds with the ATP-binding site of EGFR, enhancing its binding affinity and effectively inhibiting EGFR activation, thereby exerting its anticancer activity. Simultaneously, the methoxyethoxy group attached to the other carbon atom contributes to improving the overall binding affinity of Erlotinib to its target, which is crucial for its anti-lung cancer efficacy. Notably, these three highly attended carbon atoms not only constitute the key structural scaffold of the quinazoline ring but also connect the important functional groups, the (3-ethynylphenyl)amino and methoxyethoxy groups, highlighting our model’s ability to accurately capture critical structural features. Similarly, in our attention weight analysis of the Refametinib molecular structure (Fig 4E and 4F), the model highly attended to the nitrogen atom within the sulfonamide moiety. This nitrogen atom serves as a bridge, connecting the aromatic compound portion to the part containing the sulfonamide functional group, fusing two moieties with different chemical natures and functions into a single molecular entity. During the interaction between the drug and its biological target, this nitrogen atom may provide the ability to establish critical connections or influence the spatial structure of the molecule. It likely determines, to some extent, the affinity and specificity of the molecule toward a particular receptor or enzyme. The aromatic compound portion contains multiple substituents (e.g., fluorine, iodine, and methoxy groups), which can modulate the electronic distribution of the aromatic rings, thereby affecting the interactions, including affinity and specificity, with the target receptor. The other part contains the sulfonamide functional group, a cyclopropane ring, and a specific stereochemical diol structure. The presence of the sulfonamide and diol moieties enhances the water solubility of the molecule, which may significantly impact its absorption, distribution, metabolism, and excretion, thus playing a crucial role in its pharmacokinetic properties. Simultaneously, the presence of the cyclopropane ring not only stabilizes the overall molecular structure but also significantly influences its spatial conformation and drug activity. Therefore, the model highly attends to the critical molecular substructures of the drug molecules, which may represent key determinants influencing the specific drug efficacy of targeted therapeutics.

### Drug sensitivity prediction analysis

**Fig 5:**
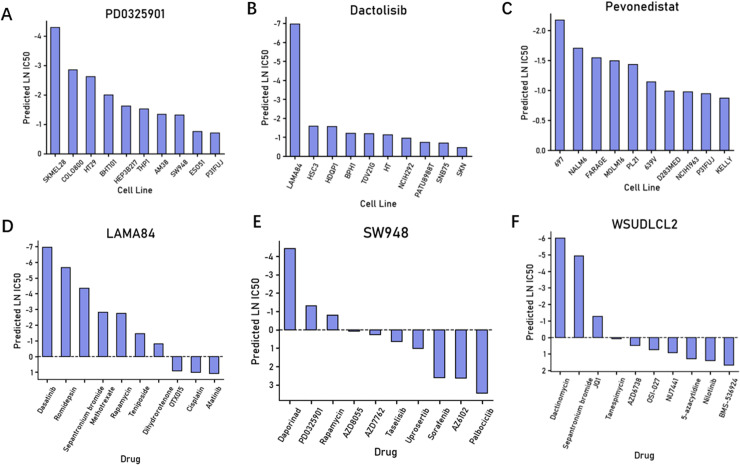
Drug efficacy analysis (A-C) Bar plots showing the predicted results for the top ten most sensitive cell lines to the three drugs PD0325901, Dactolisib, and Pevonedistat, respectively. (D-F) Bar plots showing the predicted results for the top ten most effective drugs in the three cell lines LAMA84, SW948, and WSUDLCL2, respectively.

Based on the prediction results of the PASO model, we further showcase the predictions of the top ten most sensitive cell lines to different drugs, as well as the predictions of the top ten most sensitive drugs for certain cell lines. For instance, in the model prediction results presented in Fig 5A, we can observe that SKMEL28 and COLO800, two skin melanoma cell lines, and the BHT101 thyroid cancer cell line exhibit higher sensitivity to PD0325901. Indeed, PD0325901 (Mirdametinib) is a potent non-ATP competitive MEK inhibitor, effectively inhibiting the proliferation of thyroid tumor cells or melanoma cells [52,53]. Additionally, as shown in the data presented in Fig 5B, LAMA84, the sole representative of chronic myeloid leukemia (CML) among these ten cell lines, displays more pronounced sensitivity to Dasatinib compared to the other cell lines. Dasatinib (Sprycel) is a BCR-ABL tyrosine kinase inhibitor primarily used for the treatment of chronic myeloid leukemia (CML) [54,55].

Furthermore, based on the model’s predictions of the top ten most sensitive drugs for certain cell lines, Daporinad, PD0325901, and Rapamycin show significant efficacy against the colorectal cancer (COAD) cell line SW948 (Fig 5E). Daporinad (also known as FK866 or APO866) is a NAMPT inhibitor that indirectly affects the activity of NAD+ dependent sirtuins by reducing NAD+ generation, thereby influencing cellular metabolism and growth. It shows promise in inhibiting the proliferation of colorectal cancer cells, including SW948 [56,57]. PD0325901, a MEK1/2 inhibitor, has been demonstrated to suppress the proliferation of colorectal cancer cell lines [58,59]. Rapamycin, an mTOR inhibitor, has been extensively studied for its therapeutic effects in colorectal cancer [60,61]. Additionally, the results indicate high sensitivity of the DLBCL cell line WSUDLCL2 to Dactinomycin, Sepantronium bromide, and JQ1 (Fig 5F). Dactinomycin, an antitumor antibiotic, inhibits tumor cell growth by intercalating with DNA and interfering with RNA synthesis. Studies have shown its ability to suppress proliferation and induce apoptosis in various cancer cells [62,63]. Sepantronium bromide (YM155), a survivin inhibitor, induces apoptosis and inhibits proliferation in DLBCL cell lines by downregulating survivin expression [64]. JQ1, a BET protein inhibitor, disrupts the interaction between BET proteins and transcription factors. Research has demonstrated its significant inhibitory effect on proliferation and induction of apoptosis in multiple DLBCL cell lines [65].

The model’s predictions highly align with authoritative research findings or clinical experimental data, validating the effectiveness and accuracy of our model. For those prediction results that lack sufficient literature support or clinical data verification, the model provides valuable hypotheses with research significance or medical implications.

**Fig 6:**
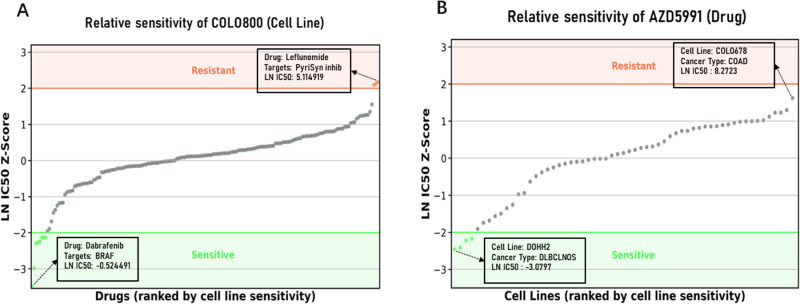
Visualization analysis of drug efficacy (A) Displays the distribution of relative sensitivity of the COLO800 cell line to various drugs. Each data point corresponds to the response of the COLO800 cell line to a specific drug, evaluated and classified based on the Z-score of ln IC50. Drugs with a Z-score less than -2 are defined as sensitive drugs for this cell line and marked in green, while those with a Z-score greater than 2 are considered drugs to which the cell line exhibits resistance and marked in orange. (B) Shows the distribution of relative sensitivity of different cell lines to the drug AZD5991, where each data point corresponds to the result of the reaction between AZD5991 and a specific cell line.

We also provide a novel perspective for visualizing drug sensitivity analysis, focusing on individual cell lines or drugs. Taking the human melanoma cell line COLO800 as an example (Fig 6A), we evaluated and classified the drugs based on their LN IC50 Z-scores. Drugs with Z-scores less than -2 were defined as sensitive drugs, while those with Z-scores greater than 2 were considered resistant drugs (following the criteria used by the GDSC). Accordingly, we report the predicted LN IC50 values for the sensitive and resistant drugs against the COLO800 cell line, along with their targeted pathways (see Table 2). The results show that the predicted LN IC50 values for the sensitive drugs are significantly lower than those for the resistant drugs, especially for drugs targeting the ERK MAPK signaling pathway. This suggests that the sensitive drugs for the COLO800 cell line primarily act on the ERK MAPK signaling pathway.

**Table 2:** Resistant & sensitive drug info on COLO800.

Drug Name	Target Pathway	Type	Predicted LN IC50	Truth LN IC50
Leflunomide	DNA replication	Resistant	5.1149	7.0213
PF-4708671	PI3K/MTOR signaling	Resistant	3.9870	5.5805
GSK269962A	Cytoskeleton	Resistant	3.9767	5.5469
Selumetinib	ERK MAPK signaling	Sensitive	0.5526	−0.4257
Doramapimod	JNK&p38 signaling	Sensitive	4.6317	2.4055
SCH772984	ERK MAPK signaling	Sensitive	−0.9456	−1.4258
Trametinib	ERK MAPK signaling	Sensitive	−4.5739	−4.8472
PD0325901	ERK MAPK signaling	Sensitive	−2.5488	−3.5265
SB590885	ERK MAPK signaling	Sensitive	1.7225	1.2269
PLX-4720	ERK MAPK signaling	Sensitive	0.1041	−0.1854
Dabrafenib	ERK MAPK signaling	Sensitive	−0.5245	−2.1871

Moreover, we analyzed AZD5991, a highly selective Mcl-1 inhibitor. Designed to target the Mcl-1 protein and induce cancer cell apoptosis, it has shown potential therapeutic effects across various cancer types, particularly for certain hematological malignancies such as acute myeloid leukemia (AML), mantle cell lymphoma, and diffuse large B-cell lymphoma (DLBCL) [66]. We applied the same Z-score rule to distinguish cell lines sensitive and resistant to AZD5991 and reported their predicted LN IC50 values (Fig 6B). Our findings are consistent with previous studies, demonstrating that cell lines exhibiting high sensitivity to AZD5991 are primarily diffuse large B-cell lymphoma (DLBCL) and acute myeloid leukemia (AML) (see Table 3), highlighting the accuracy of our model in predicting drug sensitivity and its potential applications in scientific research.

**Table 3:** Resistant & sensitive cell line Info on AZD5991.

Cell Line	Cancer	Type	Predicted LN IC50	Truth LN IC50
A4FUK	DLBCLNOS	Sensitive	−2.5607	−2.3474
KASUMI1	AML	Sensitive	−1.3434	−2.4629
DOHH2	DLBCLNOS	Sensitive	−3.0797	−3.0080
A3KAW	DLBCLNOS	Sensitive	−2.3482	−3.1395

### Predictability of clinical response in patients

To validate the clinical applicability of the model, three types of data were obtained from the TCGA database, including clinical information, gene expression profiles, and drug response records. After screening, we acquired 3,393 patient-drug combinations, encompassing 30 cancer types, 1,521 unique patients, and 142 drugs, which were then used to perform clinical drug response prediction. For gene expression profiles from patients, we utilized TPM values and applied a log(TPM+1) transformation, subsequently converting the expression profiles into pathway-based gene expression difference features (see Methods). Drug information from the response records was converted to SMILES representation through PubChemPy and then processed into 256-dimensional numerical encodings using pytoda (see Methods). The clinical drug responses in the records were categorized into four types: Complete Response (CR), Partial Response (PR), Stable Disease (SD), and Progressive Disease (PD). CR indicates complete regression of tumor lesions, PR represents significant tumor volume reduction (≥30%), SD indicates relatively stable tumor size, and PD signifies disease progression (tumor growth ≥20% or emergence of new lesions) [67]. These evaluation criteria reflect treatment outcomes ranging from the best to the worst.

**Fig 7:**
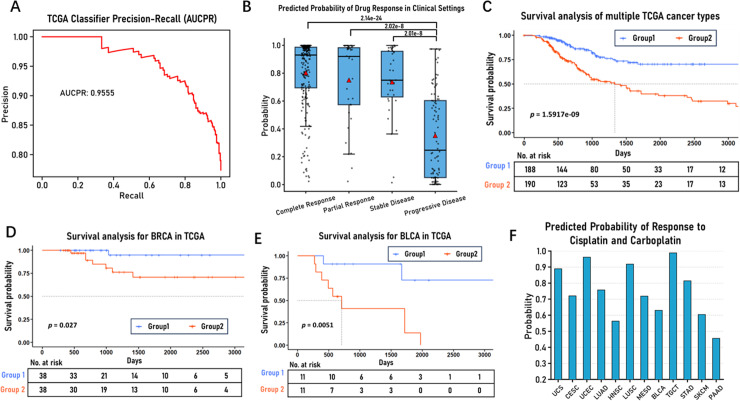
Evaluation of PASO-TCGA-Classifier model efficiency (A) Precision-recall curve representing the performance of the PASO-TCGA-Classifier model on the TCGA test dataset. (B) Boxplot displays the distribution of predicted drug response probabilities for different clinical drug responses on the TCGA test dataset. (C) Survival analysis of the TCGA test dataset across multiple cancer types. Patients were classified into two groups based on the median value of the predicted probability. Kaplan-Meier analysis was performed, and the log-rank test yielded a p-value of 1.59e-9. (D-E) Survival analysis of the TCGA test dataset for BRCA and BLCA. (F) Bar chart displays the predicted responses of Cisplatin and Carboplatin across different cancer types on the TCGA test dataset.

In drug response prediction, considering the four categories of clinical treatment responses mentioned above, we implemented a binary classification approach: PD was defined as the non-response group (label 0), while CR, PR, and SD were defined as the response group (label 1). This classification aligns with the clinical standard of using disease control as a measure of treatment effectiveness. We modified the final layer of our model with a sigmoid function to output the probability of patient response to specific drugs (range from 0 to 1), where higher response probabilities indicate patients are more likely to derive clinical benefit from the drug, and the modified model is named by PASO-TCGA-Classifier. Using the Mixed Set data splitting strategy (mentioned in the Performance section of Results), our model achieved an AUC-PR (Area Under the Precision-Recall Curve, an important metric for evaluating binary classification model performance) value of 0.9555 on the test set, as shown in Fig 7A. In-depth analysis revealed that the model’s predicted drug response probabilities showed high consistency with the four actual clinical outcomes (CR, PR, SD, PD), as illustrated in Fig 7B, with predicted probabilities being highest in the CR group, followed by the PR group, then the SD group, and the lowest in the PD group, with significant differences observed between the PR/CR/SD groups and the PD group. Additionally, we divided patients into high and low response probability groups (group1 and group2) based on the median prediction probability. When analyzing the distribution of cancer stages between these groups, we found that early-stage (stage I/II) patients were notably more prevalent in the high response probability group, while late-stage (stage III/IV) patients were significantly more common in the low response probability group, suggesting that disease stage might be one of many factors influencing drug response, as shown in S4 Fig. To assess the prognostic value of the model’s predictions, we performed Kaplan-Meier analysis on the two groups, as shown in Fig 7C, which revealed significant differences in Overall Survival (OS) between the groups (log-rank test, *p=1.59e-9*). This difference was also observed within specific cancer types, such as BLCA and BRCA, as detailed in Fig 7D and 7E. We also disclosed drug response results for different drugs within the same cancer type. In BRCA cancer, the model predicted highest response probabilities for Taxotere, Taxol, and Cytoxan (shown in S5 Fig), consistent with clinical outcomes [68,69]. Furthermore, we selected two platinum-based drugs, Cisplatin and Carboplatin, to demonstrate their predicted responses across different cancer types, as shown in Fig 7F. The figure illustrates that TGCT shows the highest response probabilities to these platinum drugs, and these predictions showed considerable alignment with clinical observations [70].

This study constructed a reliable drug response prediction model by integrating gene expression profiles, drug response records, and clinical information from the TCGA database. The model’s predictions were validated at multiple levels. Predicted probabilities showed significant correlation with actual clinical efficacy evaluations (CR, PR, SD, PD), which indicates that the model can effectively capture treatment outcomes. Moreover, prediction results effectively differentiated patient prognosis, thereby demonstrating its valuable prognostic utility. In addition, high-response drugs identified by the model aligned with clinical treatment experience, further supporting its practical relevance. Taken together, these findings confirm the model’s reliability and validate its potential value in clinical applications.

## Discussion

In this study, we developed an advanced deep learning model called PASO, which integrates the pathway-based differences values of multi-omics data with the SMILES chemical structure information of drug molecules, enabling accurate prediction of cancer cell line responses to anticancer drugs. To comprehensively evaluate the model’s performance and generalization ability, we employed three different data splitting strategies and specifically assessed our proposed method’s performance in predicting drug log-transformed IC50 values through cross-validation on the curated GDSC2 dataset. The results demonstrate that the PASO model exhibits outstanding performance, outperforming other recently proposed methods in terms of prediction accuracy.

The innovative aspect of this study lies in employing two statistical methods, the Mann-Whitney U test and the Chi-square-G test, to extract the differences between within and outside biological pathways from various omics data, utilizing these pathway-based difference values to represent the multi-omics features of cancer cell lines. The Mann-Whitney U test was employed to process gene expression data, while the Chi-square-G test was applied to handle copy number variations and gene mutation data. By combining these two methods, we were able to more comprehensively and accurately capture and analyze the pathway-level biological changes in gene expression and other omics data. Through considering a diverse set of biological pathways, we may better understand the complexities of cancer treatment and provide more comprehensive and personalized therapeutic strategies for patients. To enable the model to learn directly from chemical structures, we utilized the SMILES information as drug features, processing it into uniform-length digital encodings using pytoda. We then re-encoded the SMILES drug information through an Embedding network, multiscale convolutional neural networks, and transformer encoder, providing multi-perspective representations of drug chemical structural features, allowing the model to comprehensively learn these features and capture the intrinsic properties of molecules. By incorporating an attention mechanism, the model could identify and assign different importance weights to various biological pathways and chemical structures, thereby providing insights into the underlying biological and chemical processes behind the predictions, enhancing the overall interpretability of the model.

A significant advantage of the PASO model lies in its excellent interpretability, stemming from its SMILES & Omics Attention Network, which can compute the complex interactions between cell lines and drugs. Pathway attention analysis revealed that the model assigned higher attention scores to cancer-related signaling pathways such as TGFB, PI3K, and Notch (Fig 3A). Among the pathways with higher attention scores, cancer-related pathways occupied a higher proportion, indicating the model’s ability to automatically discover and highlight relevant cancer pathways based on the tumorigenic features of cell lines (Fig 3B). Furthermore, the model could distinguish pathway attention patterns across different types of tumor cell lines (Figs 2E and 3C) and identify signaling pathways closely associated with the targeted mechanisms of specific drugs (Fig 3D). At the molecular structural level, the model highly attended to critical substructures of the Erlotinib molecule, such as multiple carbon atoms within the core quinazoline scaffold, which are connected to important functional groups (Fig 4C), as well as the key nitrogen atom bridging the sulfonamide and aromatic moieties in the Refametinib molecule (Fig 4F). These critical substructures may influence the binding affinity and specificity of drugs to their biological targets, playing a crucial role in exerting anticancer activity. Moreover, in drug response analysis, the model’s predictions highly aligned with authoritative research findings or clinical experimental data, such as Dasatinib exhibiting the most pronounced sensitivity against chronic myeloid leukemia (CML) cell lines (Fig 5B). These results further validate the reliability of our model.

Our method still has some limitations. First, although utilizing multiple omics data, we lack other layers of data such as proteomics and metabolomics. Integrating more comprehensive biological data would provide deeper insights into cancer biology [71]. Secondly, despite improving predictive performance by fusing multi-omics data and the attention mechanism, the model complexity and number of parameters grow exponentially with increasing data. This not only makes model training more challenging and reduces robustness but also affects the interpretability of the model. Although the attention mechanism offers some explanatory power, weight allocation when handling high-dimensional data may be inaccurate, diminishing interpretability. Therefore, we need to explore new model architectures and attention mechanisms to integrate multi-omics data more efficiently while maintaining good interpretability and boosting predictive performance. Thirdly, we calculated the differences between within and outside biological pathways using statistical methods and used these pathway difference values as features instead of single-gene features. This feature engineering approach based on pathway differences achieved decent predictive effects. In addition to using the difference features, we could further analyze pathway-level biological changes from multiple angles and combine other feature engineering methods. In the future, we could incorporate pathway topology, protein interaction networks, transcription factor regulation, and other multi-faceted information into feature engineering to provide richer biological prior knowledge for the predictive model [72–74]. Finally, due to the limited amount of cancer drug response data used in the study, the model struggled to comprehensively capture cell line heterogeneity. For some targeted therapies, the lack of sufficient sample data significantly impacted the model’s predictive accuracy. Thus, in future work, we can integrate more comprehensive cancer drug response databases and clinical data to expand the sample size. Clinical data can simulate the effects of factors such as tumor microenvironment and immune responses on drug response. Such improvements would not only enhance the model’s predictive capability in cell line studies but also facilitate its validation and optimization in clinical settings, ultimately promoting its translation into practical clinical applications.

## Materials and methods

### Data preparation

To validate our proposed method, first, we downloaded gene expression profiles, gene copy number variations, and gene mutation data of cell lines from the Cancer Cell Line Encyclopedia database (CCLE, https://depmap.org/portal/download/all/). The gene expression profiles included transcripts per million (TPM) expression values for 19,194 protein-coding genes across 1,450 DepMap cell lines, which were log2-transformed after adding a pseudocount of 1. The gene copy number variation data contained copy number ratios for 25,368 genes (at the gene level) across 1,758 DepMap cell lines, which were log2-transformed after adding a pseudocount of 1. The gene mutation data summarized the damaging mutation status of 17,346 genes across 1,814 DepMap cell lines, with 0 indicating no mutation, 1 indicating the presence of a mutation but with a frequency < 0.95, and 2 indicating the presence of a mutation with a frequency ≥ 0.95. Secondly, we downloaded the c2_kegg_medicus canonical pathway gene set collection (MSigDB v2023.2.Hs) comprising 619 gene sets from the Molecular Signatures Database (MSigDB, https://www.gsea-msigdb.org/gsea/msigdb/human/collections.jsp).

In addition, we obtained drug response information for 967 cell lines to 297 drugs from the GDSC2 dataset of the Cancer Drug Sensitivity Genome (GDSC, https://www.cancerrxgene.org/downloads/drug_data). The chemical structure information of these molecular compounds was retrieved based on the SMILES presentation from PubChemPy (https://github.com/mcs07/PubChemPy). However, SMILES is not applicable to all molecular compounds. Additionally, to account for the incomplete genomic data of cell lines in the Cancer Cell Line Encyclopedia (CCLE), we ultimately selected 688 cell lines and 233 drugs, resulting in a total of 14,122 cell line-drug pairs.

Finally, the clinical data used in our study were sourced from the Genomic Data Commons data portal (GDC, https://portal.gdc.cancer.gov/) from the TCGA (The Cancer Genome Atlas) program. We downloaded three types of data: clinical data, gene expression data, and clinical drug response records. The clinical data provided comprehensive patient information, including demographic characteristics, TNM stage, pathological stage, survival status, and overall survival time. The gene expression data provided expression values for 60,660 genes for each patient, presented in multiple formats, including raw read counts, FPKM, and TPM. In this study, we used TPM values. The clinical drug response data recorded detailed drug intervention information, including drug usage, treatment duration, and treatment outcomes. After filtering, we ultimately obtained 3,393 patient-drug pairs, involving 1,521 unique patients, 142 drugs, and 30 cancer types.

### Feature extraction

#### Gene expression feature extraction.

We adopted the differences in gene expression between genes within and outside specific pathways to characterize the gene expression features of cell lines. The Mann–Whitney U test is a non-parametric method used to determine whether two independent samples originate from the same continuous distribution. Its fundamental principle involves comparing the ranks of observations between the two samples to assess whether the locations of the population distributions differ [75]. Consequently, we employed this test to compute the differences in gene expression between genes within and outside gene sets. We utilized the c2_kegg_medicus canonical pathway collection comprising 619 gene sets, ultimately constructing 619 pathway difference features for each cell line sample. The specific construction method is as follows:

For a given cell line, we denote the gene expression data within a pathway as set $A=\left\{a_{1},a_{2},\ldots ,a_{n}\right\}$ , and the gene expression data outside the pathway as set $B=\left\{b_{1},b_{2},\ldots ,b_{m}\right\}$. Here, the element $a_{n}$ in set *A* represents the observed value of the *n*-th gene within the pathway, and the element $b_{m}$ in set *B* represents the observed value of the *m*-th gene outside the pathway. Then, the two Mann–Whitney U statistics, $U_{1}$ and $U_{2}$, representing the comparison results between the gene observed values in sets *A* and *B*, can be calculated using the following formulas:


$$\begin{array}{c} U_{1}=R_{1}-\frac{n\left(n+1\right)}{2} \end{array}$$ (1)

$$\begin{array}{c} U_{2}=nm-U_{1} \end{array}$$ (2)

where $R_{1}=\overset{n}{\underset{i=1}{\sum }}rank\left(a_{i}\right)$ is the sum of ranks of all observed values in set *A*, and $rank\left(a_{i}\right)$ represents the rank of the *i*-th observed value in set *A* within the combined sample comprising sets *A* and *B*, where all observed values are assigned ranks.

Due to the varying number of genes in pathways, the majority of gene sets have a sufficient number of genes, causing their U-statistics to approximate a normal distribution. We utilize the Asymptotic Method to calculate the differences in gene expression within and outside of these gene sets. Meanwhile, for gene sets with a limited number of genes, we use the Exact Method to perform the calculations.

When the number of genes within the pathway is sufficiently large, we employed the Asymptotic Method, which is based on the large sample assumption that the distribution of the Mann-Whitney U statistic approximates a normal distribution. This method allows us to estimate the p-value using the standard normal distribution, as calculated below:


$$\begin{array}{c} p=2\times SF_{Normal}\left(\frac{U-\mu_{U}}{\sigma_{U}}\right) \end{array}$$ (3)

where $U=\max\left(U_{1},U_{2}\right)$. Under the null hypothesis, the expected value of the U statistic is $\mu_{U}=\frac{nm}{2}$, and the standard deviation is $\sigma_{U}=\sqrt{\frac{nm\left(n+m+1\right)}{12}}$, with N=n+m. By standardization, we obtain the z-value, which measures the deviation of the U statistic from its expected value and is used to assess statistical significance. $SF_{Normal}$ is the survival function of the standard normal distribution, used to calculate the probability of a random variable exceeding the z-score. This calculation method is employed to evaluate the extent to which the observed statistic deviates from the mean in the normal distribution, thereby determining the significance of the statistical result.

When the number of genes within a pathway is relatively few, we employ the Exact Method to calculate the p-value, which is computed as follows:


$$\begin{array}{c} p=2\times SF_{exact}\left(U\right) \end{array}$$ (4)

where $U=\max\left(U_{1},U_{2}\right)$, $SF_{exact}$ is the survival function applicable for small sample sizes, and $SF_{exact}\left(U\right)$ represents the probability that a random variable takes on a value greater than or equal to the statistic U under the null hypothesis. This computational method does not require assumptions about the data following any specific parametric distribution. Instead, it is based on the exact probability distribution of all possible values of the U statistic. This approach can provide more accurate p-value calculations, thereby yielding more reliable statistical inference results.

To obtain a more effective feature representation, we took the absolute value of the base-10 logarithm of the p-values, resulting in the pathway-based gene expression difference values. The magnitude of these values reflects the degree of differences in gene expression levels between genes within and outside the pathways. A larger difference value indicates a more pronounced discrepancy in gene expression between genes within and outside of the pathway. The gene expression difference features obtained through the aforementioned steps aim to capture the ability to represent alterations in cellular processes under specific biological pathways and provide more informative gene expression features as input to the model.

#### Gene mutation feature extraction.

Due to the discrete nature of gene mutation data, the aforementioned method could not be applied. Consequently, we adopted a Chi-square-G test approach, which is consistent with our strategy for processing gene expression data, namely, exploring the differences in mutations between genes within and outside specific pathways. The detailed processing steps for gene mutation data are as follows: First, we tallied the occurrences of damaging mutations in pathway gene sets within and outside cell lines, constructing a contingency table as shown in Table 4. Subsequently, we employed the Chi-square-G test method to calculate the deviation between the observed and expected frequencies based on this contingency table, obtaining the chi-square value. The chi-square value represents the magnitude of the difference in gene mutations between genes within and outside the gene set in cell lines. A larger chi-square value implies a greater difference in gene mutation occurrences between within and outside of the gene set. Ultimately, we utilized the chi-square statistic obtained through this process as the gene mutation feature for cell lines.

**Table 4:** Gene mutation contingency table.

Gene location	Gene property	
Normal	Mutant
Within gene set	a	b
Outside gene set	c	d

a, b, c, and d represent the number of genes corresponding to each case, respectively.

#### Gene copy number feature extraction.

Similar to the method used for processing gene mutation data, we also employed the Chi-square-G test to handle gene copy number data, with the following specific steps: First, we converted the copynumberratio to the absolutecopynumber using the formula $absolutecopynumber=ploidy*\left(copynumberratio\right)$, and rounded the result to the nearest integer. Here, ploidy refers to the chromosomal copy number in a cell, reflecting the copy number of a specific chromosome [76]. It is well established that the normal gene copy number on human chromosomes is 2, with values greater than 2 indicating gene amplification and values less than 2 denoting gene deletion. Therefore, we recorded genes with an absolute copy number greater than or equal to 3 as amplified, those less than or equal to 1 as deleted, and those equal to 2 as normal. Subsequently, we counted the number of genes exhibiting these three types of copy number alterations within and outside the given gene set for each cell line, constructing a contingency table similar to Table 5. Finally, we employed the Chi-square-G test method to calculate the deviation between the observed and expected frequencies based on the contingency table, obtaining the chi-square statistic. The chi-square statistic represents the overall deviation between the two groups, with a larger value indicating a more pronounced difference in copy number variations between genes within and outside of the gene set. Ultimately, we utilized this chi-square statistic as the gene copy number feature for cell lines.

**Table 5:** Copy number contingency table.

Gene position	Gene property	
Normal	Deletion & duplication
Within gene set	a	b
Outside gene set	c	d

a, b, c, and d represent the number of genes corresponding to each case, respectively.

#### Chi-square-G Test.

The Chi-square-G Test method previously employed was aimed at investigating the differences in gene mutation data and gene copy number variation data between genes within and outside the pathway gene sets. We attempted to use the chi-square test, but considering the drawbacks of the Pearson’s chi-square test when expected frequencies are low, to overcome the issue of decreased approximation accuracy of the chi-square test due to the complex distribution of pathway gene set data [77], we adopted a combination of the Pearson’s chi-square test [78] and the G-test [79], which we termed the Chi-square-G test. The computation of this method is based on the contingency tables constructed from the aforementioned two omics data types.

In most cases, the expected frequencies in the expected frequency matrix are typically greater than 5. Under this condition, it is appropriate to employ Pearson’s chi-square test to calculate the chi-square statistic. The formula for Pearson’s chi-square test is as follows:


$$\begin{array}{c} \chi^{2}=\overset{k}{\underset{i=1}{\sum }}\frac{{\left(O_{i}-E_{i}\right)}^{2}}{E_{i}} \end{array}$$ (5)

where $O_{i}$ is the observed frequency of the *i* -th category; $E_{i}$ is the expected frequency of the *i* -th category, which is the expected number of occurrences for that category under the assumption of a completely random distribution; *k* is the number of categories in the contingency table. $\chi^{2}$ is Pearson’s cumulative test statistic, which asymptotically approaches a $\chi^{2}$ distribution.

Cases where the expected frequencies are less than 5 may also occur, owing to substantial differences in gene counts between genes within and outside of the pathway gene sets. In such instances, the G-test of maximum likelihood ratios is employed to calculate the approximate chi-square statistic, as excessively small expected frequencies can diminish the approximation accuracy of Pearson’s chi-square test. The G-test constructs a test statistic based on the log-likelihood ratio between two distributions, enabling a more precise examination of the discrepancy between the distributions. The formula for the G-test is as follows:


$$\begin{array}{c} G=2\overset{k}{\underset{i=1}{\sum }}O_{i}\ln\left(\frac{O_{i}}{E_{i}}\right) \end{array}$$ (6)

where the definitions of $O_{i}$, $E_{i}$, and *k* are the same as above; furthermore, the *G* statistic follows a chi-square distribution.

If the expected frequency matrix contains expected frequencies equal to zero, the result is directly set to zero.

The aforementioned method implements the selection of an appropriate independence test method under different expected frequency conditions. This not only ensures approximation accuracy but also distinguishes the special case of zero expected frequencies as a complete violation, rendering the statistic more robust. Compared to solely using Pearson’s chi-square test, our Chi-square-G Test method can better handle the complex distribution and imbalanced features inherent in genomic data.

#### Drug feature extraction.

In this study, we utilized the SMILES strings as chemical structure information for drugs. As a sequence encoding, SMILES strings encapsulate the raw information of molecular structures. We employed the pytoda tool (https://paccmann.github.io/paccmann_datasets/index.html) to construct a drug SMILES character dictionary based on the frequency of each chemical symbol’s occurrence in the SMILES strings of 233 drugs, assigning a unique integer encoding to each symbol. While preserving the original order of characters in the SMILES strings, we assigned the corresponding encoding value to each character. Since the pre-selected drug SMILES strings had a maximum length of 186, with most drug lengths concentrated around 100, we mapped the variable-length SMILES to a fixed-length 256-dimensional numerical encoding, padding the leading positions with a specific value for lengths shorter than 256. This approach enables the deep learning-based SMILES encoder to learn features directly from the drug sequences, alleviating the reliance on traditional feature engineering. Crucially, this encoding method enhances the model’s interpretability, facilitating a better understanding and explanation of the model’s predictions.

### Model architectures

The PASO model proposed in this study is a composite network constructed from a SMILES Encoding Network, a SMILES & Omics Attention Network, and a Pharmacological Response Prediction Network. The model is designed to extract features of drug molecules from different perspectives and achieve the prediction of drug responses by integrating the drug molecules (represented by SMILES) with one or multiple omics data (pathway-based differences in gene expression, copy number variation, and mutation).

### SMILES encoding network

**Fig 8:**
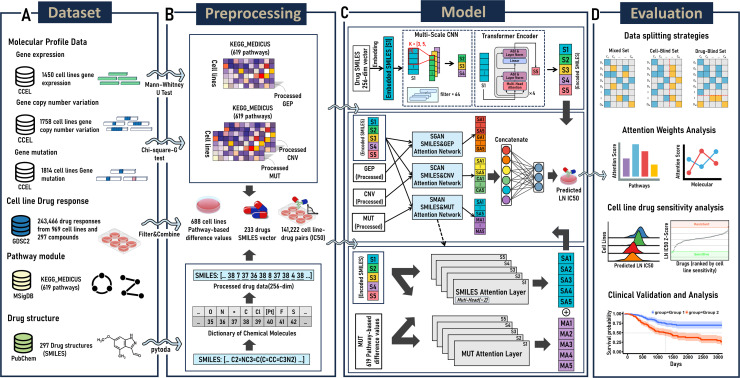
Workflow diagram (A) Data Selection: We acquired diverse datasets for training PASO from multiple databases. (B) Data Preprocessing: Statistical methods were employed to calculate the differences of various omics data within and outside biological pathways. These pathway-based difference values were utilized as cell line features. Additionally, pytoda was used to process SMILES chemical structure information. (C) Model Architecture: The model is presented in three sections, from top to bottom: The upper section illustrates the SMILES Encoding Network. The middle section depicts the overall model workflow. The lower section details the internal network structure of SMAN. (D) Evaluation of Predictions and Attention Weight Analysis: We evaluated the model using three distinct data partitioning strategies. Subsequently, we conducted drug efficacy analysis and attention weight analysis on the predicted results.


To ensure comprehensive analysis of the intricate properties of drug molecules, we constructed a SMILES Encoding Network (Fig 8C), which comprises three subnetworks: an Embedding Layer, a Multiscale CNN, and a transformer encoder. The 256-dimensional SMILES vector, preprocessed by pytoda, is first fed into the Embedding Layer, responsible for mapping the 256-dimensional SMILES vector onto a continuous embedding space [80,81]. Mathematically, this mapping process can be viewed as a transformation from a discrete space to a continuous space. The embedding transformation can be represented as:

$$\begin{array}{c} E\left(v_{i}\right)=e_{i} \end{array}$$ (7)

where *E* is the embedding function that maps the *i*-th integer encoding $v_{i}$ in the SMILES vector to its corresponding embedding vector $e_{i}$. We set the embedding dimension to 16, so $e_{i}$ is a 16-dimensional vector. Through the transformation performed by the Embedding Layer, the 256-dimensional SMILES vector is mapped to a 256×16-dimensional embedding matrix. During the training process, the model will learn this mapping, generating a corresponding continuous vector for each chemical molecule. This vector will better express the properties of the molecule, enabling the neural network to utilize this information for further analysis and prediction.

The efficacy of drugs is often associated with the activity of specific molecular substructures at the receptor binding sites [82]. Leveraging Multiscale CNN, we can effectively capture molecular substructural features of varying sizes. For instance, smaller convolutional kernels (e.g., kernel size of 3) can identify small molecular moieties such as benzene rings, while larger kernels (e.g., kernel size of 11) aid in detecting more complex heterocyclic or polycyclic structures, which are common in many drug molecules. Therefore, we employ a Multiscale CNN comprising three convolutional networks of different scales, aiming to capture the SMILES sequence features of compounds from multiple perspectives. Specifically, each independent convolutional branch convolves the SMILES embedding matrix using a convolutional kernel of a specific scale, with kernel sizes of [E, 3], [E, 5], and [E, 11], respectively, where E is the embedding dimension of SMILES from the Embedding Layer. Subsequently, a non-linear activation function is introduced, resulting in SMILES feature representations at three different scales.

In addition to employing Multiscale CNN to capture molecular substructural features at different scales, we also adopted a transformer encoder to encode long-range dependencies within the SMILES sequences [83]. The transformer encoder leverages a self-attention mechanism, enabling the model to weigh the importance of different sequence parts, regardless of their distance, thereby learning the overall properties and global features of the molecular sequences. We designed an encoder comprised of four stacked transformer encoder layers, which re-encoded the SMILES embedding matrix obtained from the embedding layer into a sequence feature representation incorporating deep contextual associations, enhancing the model’s ability to grasp global features of the drug molecular structure sequences.

The core task of the SMILES Encoding Network module is to extract features of drug molecules from different perspectives, re-encoding them into five distinct feature representations (Fig 8C), each interpreting the structure and properties of the drug molecules from its unique vantage point. These re-encoded drug features will be inputted, along with the omics features, into the SMILES & Omics Attention Network.

### SMILES and omic attention network

To effectively address the challenges of diversity and complexity in omics data, we designed the SMILES & Omic Attention Network, which comprises three sub-networks: SGAN (SMILES & GEP Attention Network), SCAN (SMILES & CNV Attention Network), and SMAN (SMILES & MUT Attention Network), dedicated to processing specific omics data and drug data (Fig 8C). This network can select the designated sub-network based on the type of input omics data, allowing for single or combined use to optimize analysis efficiency. Each subnetwork comprises five SMILES Attention Layers and five Omics Attention Layers. Every SMILES Attention Layer is responsible for interpreting the corresponding drug feature and computing the interaction between the drug feature and the omics feature, outputting a drug feature fused with the interaction information from the omics feature. Meanwhile, each Omics Attention Layer analyzes the omics feature, outputting an omics feature fused with the interaction information from the drug feature.

**Fig 9:**
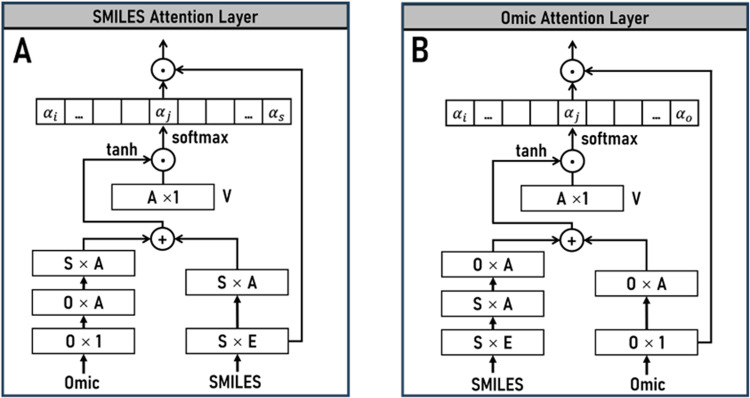
Attention network (A) The SMILES attention layer receives drug features re-encoded by the SMILES Encoding Network and preprocessed omics features, responsible for computing the complex interactions between SMILES features and specific omics features, outputting SMILES features fused with interactions with the specific omics features. (B) The Omic Attention Layer receives drug features re-encoded by the SMILES Encoding Network and preprocessed omics features, responsible for computing the complex interactions between specific omics features and SMILES features, outputting omics features fused with interactions with SMILES features.


In the domains of medical diagnostics and drug discovery, model interpretability is extremely crucial. The attention mechanism endows the model with the ability to analyze and interpret the potential biological and chemical processes underlying the predictions, thereby enhancing the model’s interpretability. Inspired by Matteo Manica et al. [10] , we designed the SMILES Attention Layer and Omics Attention Layer, neural network layers based on the attention mechanism, to model and analyze the complex interactions between drug features and omics features. The former is used to analyze drug features that have been re-encoded by the SMILES Encoding Network, while the latter is used to analyze omics data after preprocessing. Specifically, the SMILES Attention Layer, as illustrated in Fig 9A, has two inputs: the omics sequence $O\in {\mathbb{R}}^{O\times 1}$ on the left and the drug sequence $S\in {\mathbb{R}}^{S\times E}$ on the right. First, the SMILES sequence undergoes a linear mapping, projecting it into the Attention space to obtain *S*^′^:

$$\begin{array}{c} S^{\prime }=W_{s}S \end{array}$$ (8)

where $W_{s}\in {\mathbb{R}}^{E\times A}$ represents the trainable parameters used for the linear transformation of the drug sequence *S*.

Subsequently, the omics sequence undergoes two linear mappings, projecting it into the Attention space of the SMILES sequence, obtaining *O*^′^:


$$\begin{array}{c} O^{\prime }=W_{o}^{'}OW_{o} \end{array}$$ (9)

where $W_{o}\in {\mathbb{R}}^{1\times A}$ and $W_{o}^{'}\in {\mathbb{R}}^{S\times A}$ are trainable parameters, with $W_{o}$ and $W_{o}^{'}$ performing linear transformations on the omics sequence *O* sequentially.

Next, *S*^′^ and *O*^′^ are added together, and the result is processed through a tanh activation function. Then, the result is mapped from the Attention space to a one-dimensional space, enabling the use of the softmax function to normalize the sequence, yielding the weight matrix *α*:


$$\begin{array}{c} \alpha =\text{softmax}\left(\tanh\left(O^{\prime }+S^{\prime }\right)V\right) \end{array}$$ (10)

where $V\in {\mathbb{R}}^{A\times 1}$ represents trainable parameters. The tanh function constrains the sequence within the range of [-1, 1], not only endowing the model with the non-linear capability to process information but also preparing for the subsequent softmax probability allocation. The softmax function assigns weights to each chemical molecule in the SMILES sequence based on the normalized probability distribution, enabling the model to place higher attention weights on the molecularly important structures.

Finally, we perform a matrix multiplication between the weight matrix *α* and the drug sequence *S* to obtain the final drug sequence representation output:


$$\begin{array}{c} output=S⊙\alpha \end{array}$$ (11)

The computed output vector is obtained by assigning different weights $\alpha_{i}$ to each vector $S_{i}$ in the drug molecule feature sequence *S* according to its relevance with the omics feature *O*, and then performing a weighted sum. It incorporates the interaction effects between the drug molecule features and the omics features.

Similarly, the Omics Attention Layer, as depicted in Fig 9B, is dedicated to computing the interaction features of the omics feature *O* with respect to the drug molecule feature *S*. Its computation process is similar to the aforementioned method.

Through the SMILES Attention Layer and Omics Attention Layer, the drug SMILES sequence features and omics data are effectively fused, with important interactions between features being enhanced, forming a powerful combined feature representation that comprehensively reflects the complex relationship between the drug and the cell line. This provides a crucial model input for the subsequent task of predicting drug efficacy based on the cell line-drug pair.

### Pharmacological response prediction network

The Pharmacological Response Prediction Network is a neural network module specifically designed to predict drug responses (Fig 8C). It primarily consists of Batch Normalization and Multi-Layer Perceptron (MLP) [81], aiming to predict the LN IC50 values of specific drugs on cancer cell lines. The IC50 is a quantitative measure that indicates the amount of a specific inhibitory drug required to inhibit the growth of a given cancer cell line by 50% in vitro.

In the Pharmacological Response Prediction Network, the interaction features derived from the SMILES & Omic Attention Network are first concatenated to form a comprehensive feature set as input. The input interaction features are initially standardized through a Batch Normalization layer, a step intended to enhance the stability and convergence speed of model training while providing slight regularization. The standardized features are then passed to the MLP layer, which consists of weighted neurons and activation functions. This layer is responsible for capturing the complex nonlinear relationships among the input features and outputting the final prediction results.

## Supporting information

S1 Fig Comparison of difference values of gene expression between DNA replication and other pathways in SCLC cell lines. (TIF)

S2 Fig Comparison of drug chemistry at locations with PCA_1<0.002 (Left) versus PCA_1>=0.002 (Right). (TIF)

S3 Fig Boxplot of cross-validation MSE results of PASO under different combinations of omics data. (TIF)

S4 Fig Distribution of different stages across multiple TCGA cancer types. (TIF)

S5 Fig Bar chart of drug response results for different drugs in BRCA. (TIF)

S1 Table Detailed configuration of key hyperparameters across models. (PDF)
