# Supplementary figures and images for: Anticancer drug response prediction integrating multi-omics pathway-based difference features and multiple deep learning techniques
Source: PLoS Comput Biol. 2025 Mar 31;21(3):e1012905. doi: 10.1371/journal.pcbi.1012905 (PMC11978092; doi:10.1371/journal.pcbi.1012905)

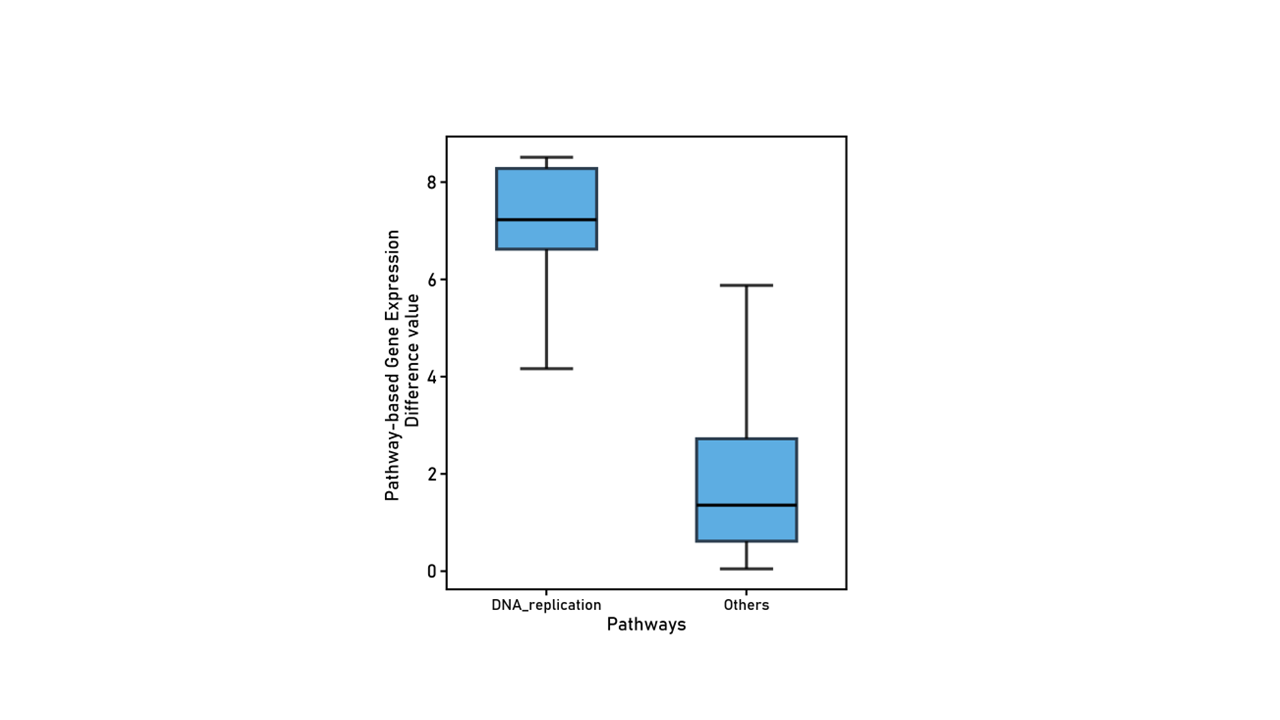

Supplement: S1 Fig — (TIF) [file pcbi.1012905.s001.tif]

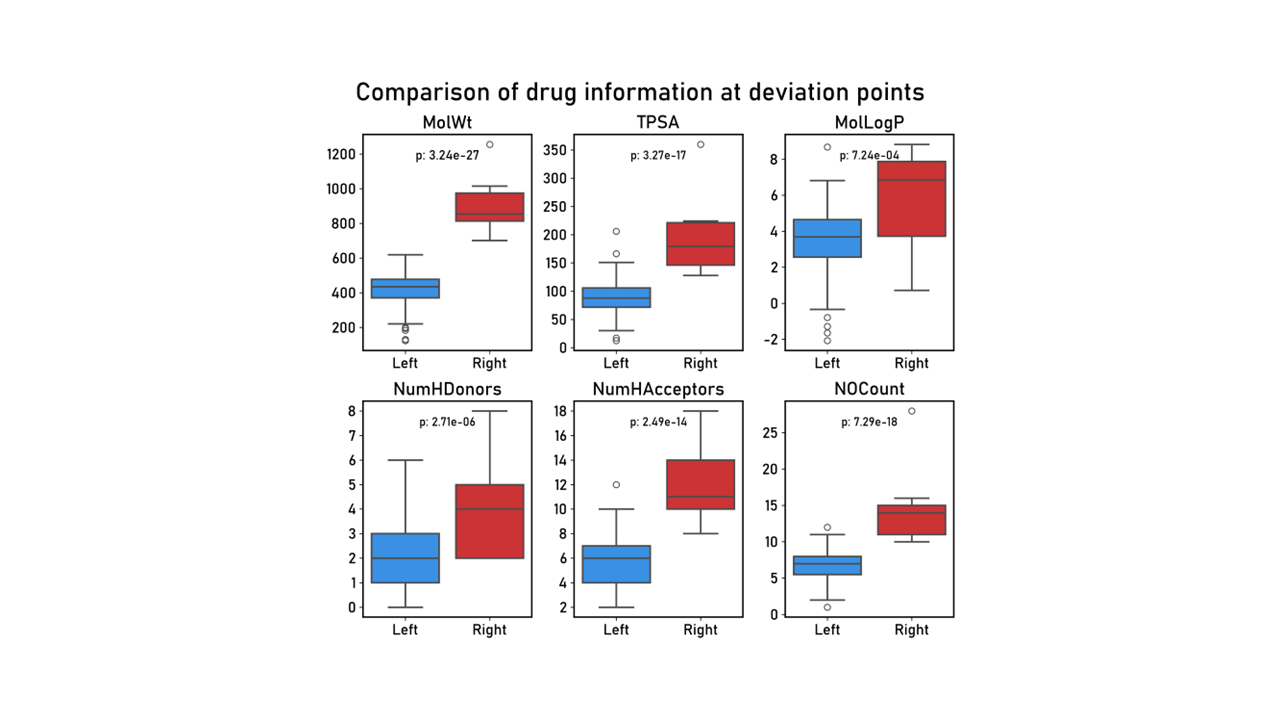

Supplement: S2 Fig — (TIF) [file pcbi.1012905.s002.tif]

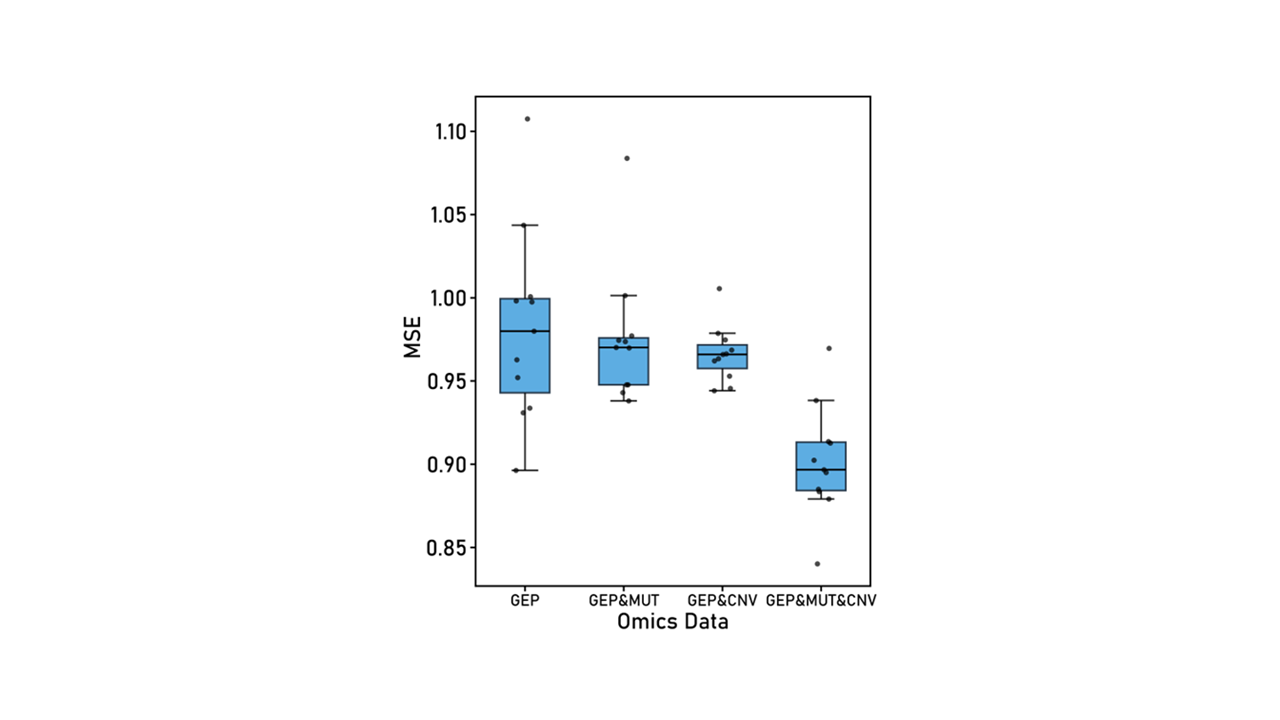

Supplement: S3 Fig — (TIF) [file pcbi.1012905.s003.tif]

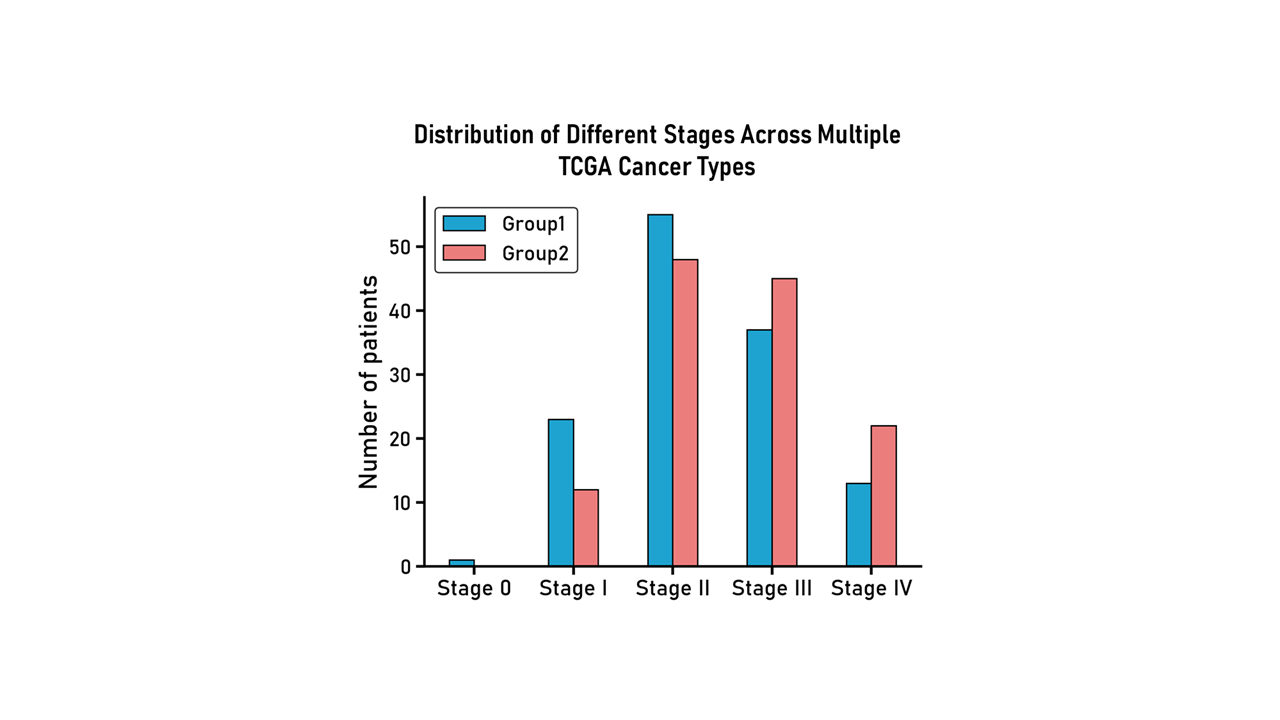

Supplement: S4 Fig — (TIF) [file pcbi.1012905.s004.tif]

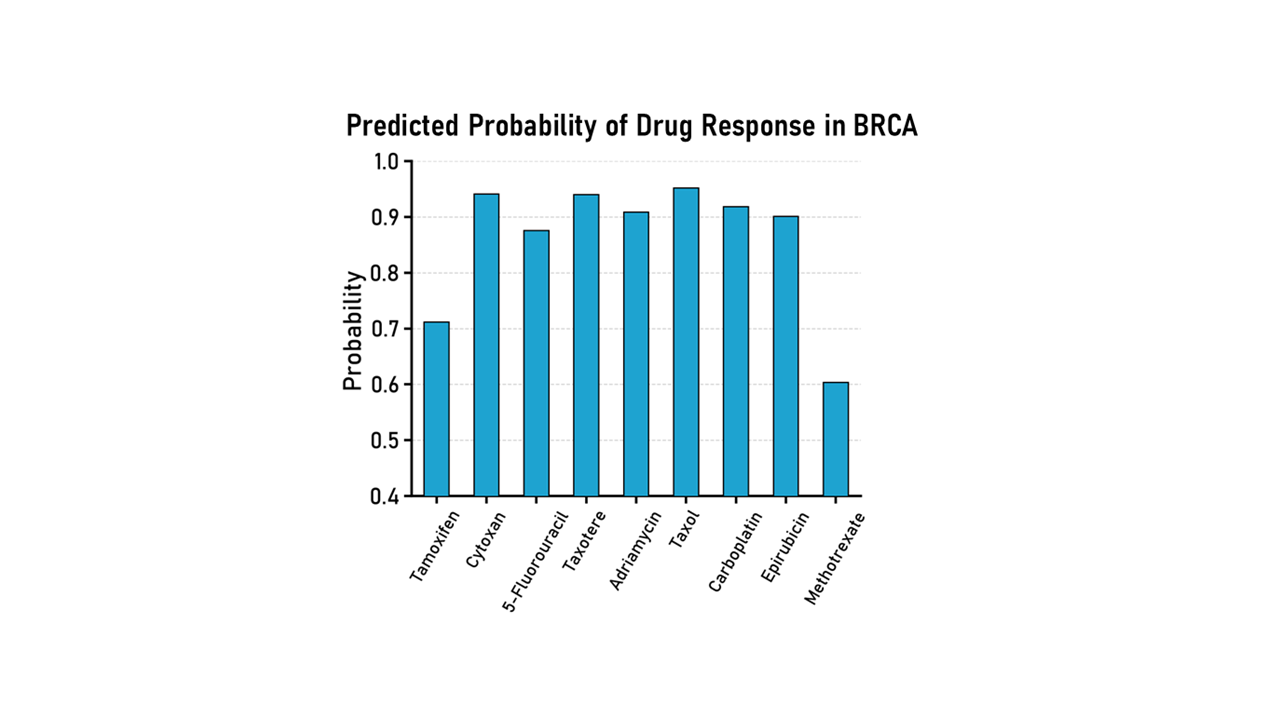

Supplement: S5 Fig — (TIF) [file pcbi.1012905.s005.tif]
