# Supplementary material for: Anticancer drug response prediction integrating multi-omics pathway-based difference features and multiple deep learning techniques
Source: PLoS Comput Biol. 2025 Mar 31;21(3):e1012905. doi: 10.1371/journal.pcbi.1012905 (PMC11978092; doi:10.1371/journal.pcbi.1012905)
Supplement: S1 Table — (PDF) [file pcbi.1012905.s006.pdf]

Anticancer Drug Response Prediction Integrating Multi-Omics Pathway-Based Difference Features and Multiple Deep Learning Techniques

S6 Table. Detailed Configuration of Key Hyperparameters Across Models

Model	Hyperparameters
Machine Learning Models	
SVM	kernel="rbf", degree=3, gamma="scale", coef0=0.0, tol=1e-3, C=1.0, epsilon=0.1, shrinking=True, cache_size=200, verbose=False, max_iter=-1,
Random Forest	n_estimators=100, criterion="squared_error", min_samples_split=2, min_samples_leaf=1, min_weight_fraction_leaf=0.0, max_features=1.0, bootstrap=True, oob_score=False, warm_start=False
LightGBM	boosting_type: 'gbdt', colsample_bytree: 1.0, importance_type: 'split', learning_rate: 0.1, max_depth: -1, min_child_samples: 20, min_child_weight: 0.001, n_estimators: 100, subsample_for_bin: 200000
XGBoost	objective: reg:squarederror, booster: None, callbacks: None, colsample_bylevel: None, colsample_bynode: None, colsample_bytree: None, enable_categorical: False
Deep Learning Models (Pytorch 2.1.0)	
Precily	n_layers: 2, dropout_rate: 0.1, learning_rate: 0.0001, units_0: 252, units_1: 88, batch_size: 512, optimizer: "adam", loss_fn: "mse"
PathDSP	n_in: 2113, n_hidden1: 864, n_hidden2: 400, n_hidden3: 180, n_hidden4: 100, drop_rate: 0.1, learning_rate: 0.001, batch_size: 512, optimizer: "adam", loss_fn: "mse"
PASO	number_of_pathways: 619, smiles_padding_length: 256, stacked_dense_hidden_sizes: [1024, 256, 64], filters: [64, 64, 64], molecule_gep_heads: [2,2,2,2,2], gep_heads: [1,1,1,1,1], smiles_embedding_size: 16, kernel_sizes: [3,16], [5,16], [11,16]], n_heads: 2, num_layers: 4, dropout: 0.3, batch_norm: true, batch_size: 512, learning_rate: 0.001, optimizer: "adam", loss_fn: "mse"

PASO (Omics)	number_of_pathways: 619, smiles_padding_length: 256, stacked_dense_hidden_sizes: [1536,256,64], filters: [64,64,64], molecule_gep_heads: [2,2,2,2,2], molecule_cnv_heads: [2,2,2,2,2], molecule_mut_heads: [2,2,2,2,2], gep_heads: [1,1,1,1,1], cnv_heads: [1,1,1,1,1], mut_heads: [1,1,1,1,1], smiles_embedding_size: 16, kernel_sizes: [3,16], [5,16], [11,16]], dropout: 0.3, batch_norm: true, batch_size: 512, learning_rate: 0.001, optimizer: "adam", loss_fn: "mse"
